# Final Results from the First European Real-World Experience on Lusutrombopag Treatment in Cirrhotic Patients with Severe Thrombocytopenia: Insights from the REAl-World Lusutrombopag Treatment in ITalY Study

**DOI:** 10.3390/jcm13133965

**Published:** 2024-07-06

**Authors:** Paolo Gallo, Antonio De Vincentis, Francesca Terracciani, Andrea Falcomatà, Valeria Pace Palitti, Maurizio Russello, Anthony Vignone, Domenico Alvaro, Raffaella Tortora, Marco Biolato, Maurizio Pompili, Vincenza Calvaruso, Veneziano Marzia, Marco Tizzani, Alessandro Caneglias, Francesco Frigo, Marcantonio Gesualdo, Alfredo Marzano, Valerio Rosato, Ernesto Claar, Rosanna Villani, Antonio Izzi, Raffaele Cozzolongo, Antonio Cozzolino, Aldo Airoldi, Chiara Mazzarelli, Marco Distefano, Claudia Iegri, Stefano Fagiuoli, Vincenzo Messina, Enrico Ragone, Rodolfo Sacco, Pierluigi Cacciatore, Flora Masutti, Saveria Lory Crocé, Alessandra Moretti, Valentina Flagiello, Giulia Di Pasquale, Antonio Picardi, Umberto Vespasiani-Gentilucci

**Affiliations:** 1 Fondazione Policlinico Universitario Campus Bio-Medico, 00128 Roma, Italy; 2 Research Unit of Internal Medicine, Department of Medicine and Surgery, Università Campus Bio-Medico di Roma, 01128 Roma, Italy; 3 Santo Spirito Hospital, ASL Pescara, 65026 Pescara, Italy; 4 Liver Unit, Arnas Garibaldi, 95124 Catania, Italy; 5 Department of Translational and Precision Medicine, Sapienza University of Rome, 00185 Roma, Italy; 6 Liver Unit, Department of Medicine, Cardarelli Hospital, 80131 Napoli, Italy; 7 Fondazione Policlinico Universitario Agostino Gemelli IRCCS, 01128 Roma, Italy; 8 Gastroenterology and Hepatology Unit, University of Palermo, 90128 Palermo, Italy; 9 Division of Gastroenterology and Hepatology, A.O.U. Città della Salute e della Scienza di Torino, University of Turin, 10126 Turin, Italy; 10 UOSD Epatologia, Ospedale Evangelico Betania, 80147 Naples, Italy; 11 C.U.R.E. (University Center for Liver Disease Research and Treatment), Liver Unit, Department of Medical and Surgical Sciences, University of Foggia, 71122 Foggia, Italy; 12 Department of Infectious Diseases, D. Cotugno Hospital, 80131 Napoli, Italy; 13 Gastroenterology Unit, National Institute of Gastroenterology, IRCCS “S de Bellis” Research Hospital, 70013 Castellana Grotte, Bari, Italy; 14 Gastroenterology Unit, Ospedale del Mare, 80147 Napoli, Italy; ancozzolino@libero.it; 15 Hepatology and Gastroenterology, ASST GOM Niguarda, 20162 Milan, Italy; 16 UOSD Epatologia-Ospedale Umberto I Siracusa-ASP 8, 96100 Siracusa, Italy; 17 Gastroenterology, Department of Medicine, University of Milan Bicocca, 20126 Milan, Italy; 18 Infectious Disease Unit, AORN Sant’Anna e San Sebastiano, 81100 Caserta, Italy; 19 UOC Medicina Infettivologica e dei Trapianti UOS Eco Interventistica, Clinica AORN Dei Colli-Ospedale Monaldi, 80131 Napoli, Italy; 20 Gastroenterology and Digestive Endoscopy Unit, Foggia University Hospital, 71122 Foggia, Italy; 21 Liver Clinic, University Hospital of Trieste (Azienda Sanitaria Giuliano-Isontina), 34149 Trieste, Italy; 22 Gastroenterology Unit, San Filippo Neri Hospital, 00135 Rome, Italy; 23 Research Unit of Clinical Medicine and Hepatology, Department of Medicine and Surgery, Università Campus Bio-Medico di Roma, 01128 Roma, Italy

**Keywords:** severe thrombocytopenia, thrombopoietin receptor agonists (TPO-RA), lusutrombopag, portal vein thrombosis (PVT)

## Abstract

**Background and aims:** Management of severe thrombocytopenia poses significant challenges in patients with chronic liver disease. Here, we aimed to evaluate the first real-world European post-marketing cohort of cirrhotic patients treated with lusutrombopag, a thrombopoietin receptor agonist, verifying the efficacy and safety of the drug. **Methods:** In the REAl-world Lusutrombopag treatment in ITalY (REALITY) study, we collected data from consecutive cirrhotic patients treated with lusutrombopag in 19 Italian hepatology centers, mostly joined to the “Club Epatologi Ospedalieri” (CLEO). Primary and secondary efficacy endpoints were the ability of lusutrombopag to avoid platelet transfusions and to raise the platelet count to ≥50,000/μL, respectively. Treatment-associated adverse events were also collected. **Results:** A total of 66 patients and 73 cycles of treatment were included in the study, since 5 patients received multiple doses of lusutrombopag over time for different invasive procedures. Fourteen patients (19%) had a history of portal vein thrombosis (PVT). Lusutrombopag determined a significant increase in platelet count [from 37,000 (33,000–44,000/μL) to 58,000 (49,000–82,000), *p* < 0.001]. The primary endpoint was met in 84% of patients and the secondary endpoint in 74% of patients. Baseline platelet count was the only independent factor associated with response in multivariate logistic regression analysis (OR for any 1000 uL of 1.13, CI95% 1.04–1.26, *p* 0.01), with a good discrimination power (AUROC: 0.78). Notably, a baseline platelet count ≤ 29,000/μL was identified as the threshold for identifying patients unlikely to respond to the drug (sensitivity of 91%). Finally, de novo PVT was observed in four patients (5%), none of whom had undergone repeated treatment, and no other safety or hemorrhagic events were recorded in the entire population analyzed. **Conclusions:** In this first European real-world series, lusutrombopag demonstrated efficacy and safety consistent with the results of registrational studies. According to our results, patients with baseline platelet counts ≤29,000/μL are unlikely to respond to the drug.

## 1. Introduction

Severe thrombocytopenia (platelet count <50,000/μL) is one of the most common complications in patients with chronic liver disease (CLD) and poses significant management challenges, particularly with regard to the risk of bleeding during invasive procedures. In recent years, new insights into the physiopathology of thrombocytopenia have led to the development of new drugs that go beyond the limits of the current standard of care [1]. Indeed, platelet transfusions have several disadvantages, including problems with availability, side effects, and short and unpredictable efficacy [2,3].

Lusutrombopag and avatrombopag are the second-generation thrombopoietin receptor agonists (TPO-RAs) approved for the treatment of severe thrombocytopenia in patients with CLD undergoing invasive procedures [1]. Lusutrombopag was launched in Japan in 2015 and endorsed in Europe in 2019 based on two randomized, placebo-controlled, double-blind studies. In L-PLUS 1, patients treated with lusutrombopag required much less frequently pre-operative platelet transfusions compared to those receiving placebo (79.2% vs. 12.5%, *p* < 0.0001), with a significantly higher increase in platelet count (77% vs. 6%, *p* < 0.0001) [4]. L-PLUS 2 confirmed the efficacy and safety of the drug for both the endpoints, with no safety concerns [5].

Notably, to date, limited post-marketing data are available, derived from retrospective analyses, sporadic case reports/series or data from administrative databases, mainly from Japan [6,7,8,9,10,11]. Real-world data are fundamental to confirm the results obtained in registry studies in the context of daily clinical practice, where patients are less selected, with potentially more severe index pathologies and with a greater number of comorbidities [12,13].

Based on this background, here we report data of efficacy and safety from the first European real-world cohort of cirrhotic patients with severe thrombocytopenia treated with lusutrombopag before undergoing invasive procedures. Our results confirm the efficacy profile of the drug in a real-world setting. Baseline platelet count emerges as the only independent predictor of response. At the same time, a previous treatment failure predicts non-response to the drug in those patients with repeated exposures. Finally, in this population, we observed a 5% incidence of portal vein thrombosis (PVT). Actually, the fact that PVT is a possible complication of cirrhosis itself or of specific invasive procedures, together with the lack of a control arm, does not allow us in any way to establish a causal link with lusotrombopag treatment. Larger series are clearly awaited to shed more light on this point.

## 2. Patients and Methods

The REAl-world Lusutrombopag treatment in ITalY (REALITY) study is a retrospective study of data collected from cirrhotic patients treated with lusotrombopag prior to planned invasive procedures. Nineteen Italian centers, mostly affiliated with the “Club Epatologi Ospedalieri” (CLEO), joined the REALITY project. The study protocol conformed to the ethical norms and standards of the Declaration of Helsinki. Ethical approval was waived by the local Ethics Committees in view of the retrospective nature of the study and of the anonymized collection of data registered during clinical activity.

Sixty-six consecutive cirrhotic patients with severe thrombocytopenia ( <50,000/μL), treated with lusotrombopag before undergoing planned invasive procedures between March 2021 and March 2023, were enrolled in the study. The endpoints were evaluated concerning the procedures rather than the patients, so it was possible to enroll the same patient repeatedly if undergoing different invasive procedures over time. Patients received the drug as per label, and lusutrombopag was administered in all cases at the dose of 3 mg daily for 7 days, starting 10–16 days before the planned procedure. Patients were excluded only if a follow-up of less than 1 month after treatment was available.

The following data were collected: etiology of CLD; baseline socio-demographic and clinical characteristics, including the use of beta-blockers, liver function tests and ultrasound parameters (splenic and portal vein diameters); type of invasive procedure; baseline platelet, leukocyte and hemoglobin counts; pre-procedural platelet and hemoglobin counts; peri-procedural transfusion requirements; post-procedural platelet and hemoglobin counts at set time points (follow-up 1: 0–48 h post-procedure; follow-up 2: 3–30 days post-procedure); and occurrence of de novo PVT or thrombosis at any site and of any other adverse event within six months (follow-up 3: 30 days to 6 months).

All the data collected were reported in a shared database and analyzed centrally. Categorical data were described as frequencies and percentages, and continuous variables were summarized as medians and interquartile ranges (IQRs).

The primary efficacy endpoint was defined as the ability of lusutrombopag to avoid platelet transfusions before and during procedures, while the secondary endpoint was defined as the ability of the drug to raise the platelet count to ≥50,000/μL before the planned procedure. Treatment-emergent adverse events were recorded and analyzed. Univariate and multivariate logistic regression analyses were performed to identify factors associated with an effective response (platelet count ≥ 50,000/μL). Individual variables entered the multivariate model if the *p*-value in the univariate analysis was < 0.1. Predictive performances were estimated by calculating the area under the receiver operating characteristic (AUROC) curve, sensitivity and specificity. All statistical analyses were performed with R statistical analysis software.

## 3. Results

### 3.1. Clinical Characteristics of the Patients

Table 1 summarizes the main socio-demographic and clinical characteristics of the study population.

Lusutrombopag was administered 73 times before a scheduled invasive procedure in 66 patients. Five subjects underwent different procedures over time, each requiring pretreatment with lusotrombopag beforehand. These five subjects underwent a total of 13 procedures, each preceded by lusotrombopag pretreatment. Therefore, our study reports data for 66 patients and 73 distinct procedures. In addition, some patients had also received the drug on previous occasions for procedures not included in our analysis because no clinical or biochemical data were collected for them. Altogether, the group of subjects with previous exposure to the drug represented 15% of the study population.

**Figure 1:**
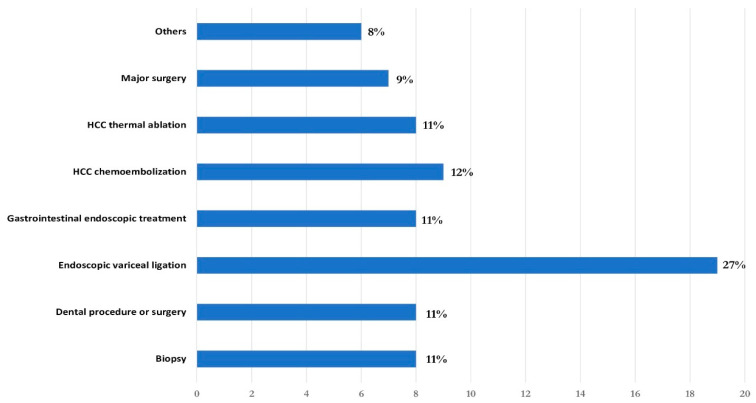
Invasive procedures performed.

The median age was 66 (IQR 56–72) years and 32% were female. A total of 45 patients (63%) had preserved liver function (Child-Pugh class A), while 20 (28%) and 6 (9%) patients were in Child-Pugh class B and C, respectively. The median body mass index (BMI) was 25 (IQR 21–28), and 27 patients (37%) had diabetes mellitus. Chronic viral hepatitis (55%), mainly HCV-related (40%), was the primary cause of CLD. The other most prevalent etiologies were metabolic dysfunction-associated (21%) and alcoholic CLD (13%). The median portal vein diameter was 14 mm (IQR 14–17), the median splenic interpolar diameter was 18 cm (IQR 16–20) (normal value < 12 cm) and forty-five patients (62%) were treated with beta-blockers for portal hypertension. Endoscopic treatments were the most common procedures (38%), with endoscopic band ligation of oesophageal varices in first place (27%) and other gastrointestinal endoscopic treatments (polypectomy and biopsy) (11%), followed by hepatocellular carcinoma (HCC) chemoembolization (12%) and radiofrequency ablation (11%), dental procedures/surgery (11%), parenchymal biopsy (11%), major intra-abdominal surgery (9%; HCC resection, cholecystectomy and esophageal resection) and others (8%; external beam radiotherapy, trans-jugular intrahepatic portosystemic shunt placement, and phacoemulsification) (Figure 1).

A total of 14 patients (19%) had a history of PVT (12 with portal cavernous transformation) (Table 2).

The median age of these patients was 72 (IQR 52–77) years and 36% were female. Nine patients (64%) had preserved liver function (Child-Pugh class A), while four (28%) and one (8%) patients were in Child-Pugh class B and C, correspondingly. HCV hepatitis (57%) was the primary cause of CLD. The median portal vein diameter was 14 mm (IQR 13–19), and the median splenic interpolar diameter was 17 cm (IQR 14–22).

Finally, in the overall population, five patients needed to use the drug repeatedly for different procedures they had to undergo over time (three patients twice and two patients thrice). Notably, in all cases, the time between the different treatments with the drug and subsequent procedures was at least 6 months. These patients had preserved liver function (Child-Pugh A6/B8), were stable over time, and only one had a history of PVT (treated twice). Lastly, one patient underwent two different consecutive procedures following the same treatment cycle with lusutrombopag.

### 3.2. Effect of Lusutrombopag

**Figure 2:**
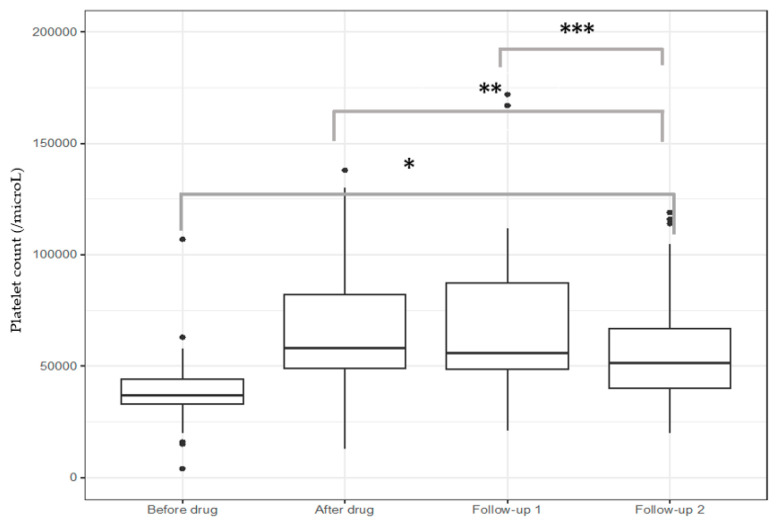
Efficacy of lusutrombopag to raise platelet count. * *p* < 0.001 vs. all groups, ** *p* < 0.01 after drug vs. follow-up 2, *** *p* = 0.003 follow-up 1 vs. follow-up 2. Follow-up 1: 0–2 days after procedures; follow-up 2: 3–30 days after procedures.

The median baseline platelet count was 37,000 (33,000–44,000/μL), and the drug induced a significant increase in platelet count before procedures [58,000 (49,000–82,000/μL), *p* < 0.001]. This increase was maintained over time, particularly within the first 2 days after the procedure [(56,000 (48,500–87,500/μL), n = 67], and slowly decreased over the next 30 days [51,499 (40,000–66,750/μL), n = 66] (Figure 2).

The primary efficacy endpoint (avoidance of platelet transfusion) was achieved in 84% of patients, while the secondary endpoint (a platelet count ≥ 50,000/μL after treatment) was achieved in 74% of treatments. Among patients who received repeated treatment with the drug, three out of five met the primary and secondary endpoints after all repeated treatments, while the other two patients failed to respond to either treatment. Among the six patients in Child-Pugh class C, four showed a response to the drug. Notably, no patient experienced post-procedural bleeding, either among those who responded to the drug, those who did not respond, or those who used the drug repeatedly.

### 3.3. Factors Associated with Achieving a Platelet Count of ≥50,000 after Treatment with Lusutrombopag

In univariate analysis (Table 3), baseline platelet count (OR of 1.13 for any 1000/uL, CI95% 1.06–1.22, *p* < 0.001) and spleen diameter (OR of 0.74 for any cm, CI95% 0.56–0.95, *p* = 0.02) were directly and indirectly associated with response to lusutrombopag treatment (as defined by platelets elevation ≥ 50,000/μL), respectively. Notably, the leukocyte count was also nearly significant (OR of 1.51 for any 1000/uL, CI95% 1.02–2.43, *p* = 0.06).

**Figure 3:**
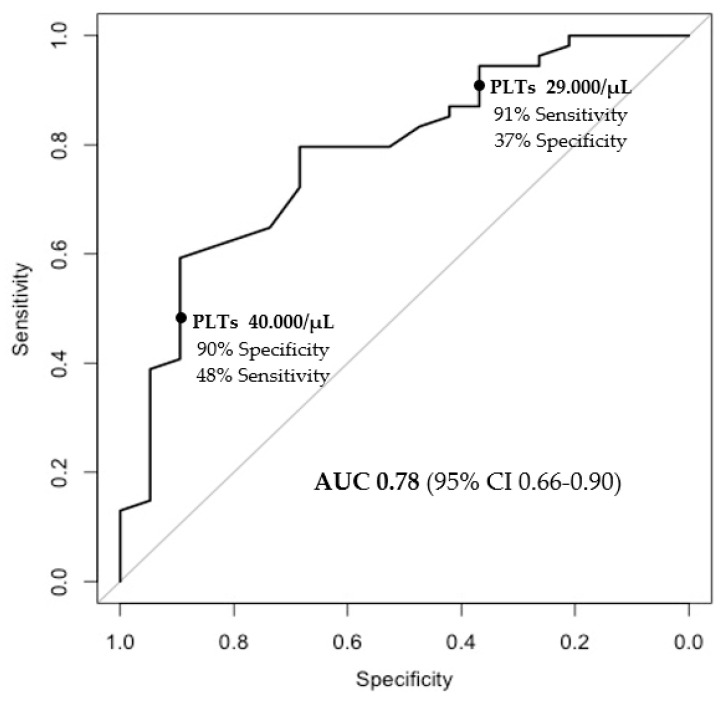
Baseline platelet values and prediction of response.

In the multivariate analysis, only baseline platelet count was independently associated with the response to lusutrombopag (OR for any 1000/uL of 1.13, CI95% 1.04–1.26, *p* = 0.01) (Table 3). Furthermore, platelet count displayed an adequate discriminatory power with respect to response to the drug, with an AUROC of 0.78 (Figure 3).

Notably, a baseline platelet count ≤29,000/μL was identified as the threshold for identifying patients unlikely to respond to the drug, with a sensitivity of 91% (Figure 3), as only 9 out of 100 patients were incorrectly classified as non-responders.

### 3.4. Safety of Lusutrombopag

Lusutrombopag was well tolerated and no patients discontinued the drug. Four patients (5%) developed de novo PVT, occurring in two patients within one month after the procedure (thermal ablation of HCC in both cases) and in others between 30 and 180 days after the procedure (endoscopic band ligation of oesophageal varices and thermal ablation of HCC). However, we have to clarify that we only have 53 follow-ups at time 3 out of the 73 patients included in the study. The maximum platelet count achieved in these patients was 62,000/μL, and only one of the four patients had a history of portal cavernous transformation and developed right main-branch portal vein thrombosis. In a multivariable analysis including anthropometric parameters, liver function, ultrasound parameters, baseline blood counts or prior use of the drug, history of previous hepatic decompensation had the most unbalanced odds ratio towards a positive association (OR 2.91, CI95% 0.35–6.05, *p* = 0.36), although far from statistical significance, likely also due to the small sample size. Furthermore, there were no thrombotic events in patients receiving repeated doses of the drug, nor any bleeding complications during or after the procedure. No other adverse events were observed during treatments. Finally, no safety concerns were observed in patients with Child-Pugh class C.

## 4. Discussion and Conclusions

This is the first study to evaluate the real-world use of lusutrombopag in a European cohort of cirrhotic patients. Our results confirm the efficacy and safety of the drug, making it a feasible option for hepatopathic patients with severe thrombocytopenia undergoing invasive procedures. In addition, baseline platelet count was identified as the only factor associated with an effective response to the drug, as defined by achieving a platelet count ≥ 50,000/μL.

Previous reports, mainly from Japan, have already extended the knowledge about the efficacy and safety of lusotrombopag to the post-marketing setting [6,7,8,9,10,11]. However, these studies were largely based on administrative reports or databases, and the patients included frequently displayed higher baseline platelet counts (>50,000/µL) and better liver function. In the present study, the efficacy and safety profiles of the drug observed in the pivotal trials were confirmed in a real-world experience database from hepatology centers. Moreover, we evaluated a highly heterogenous population of cirrhotic patients, including those with decompensated cirrhosis (Child-Pugh B and C), and with a history of PVT (excluded from registrational trials), who appear to benefit equally from the drug according to Japanese real-world post-marketing data [14]. Although the numbers are too small to draw definitive conclusions, our data suggest a potentially effective and safe biological action even in subjects with Child-Pugh class C, who were also excluded from the registrational trials.

Despite the differences between our patient population and registrational trials, the present data confirm the efficacy of lusutrombopag, with 84% of patients avoiding platelet transfusions before planned invasive procedures. It is also important to emphasize that no bleeding events were observed in patients undergoing invasive procedures after the use of lusutrombopag. However, bleeding events did not occur in patients who were transfused due to non-response to the drug either. Furthermore, the reduction in bleedings was not a study endpoint as it was not designed against a control cohort of patients allocated to not receiving the drug.

Furthermore, we found the baseline platelet count as the only predictor of the response to lusotrombopag and as potentially able to identify patients who may truly benefit from the drug. Notably, while registrational trials have not found an association between baseline platelet count and therapeutic response, a low baseline platelet count was the only predictive factor of lusotrombopag failure in previous real-world settings [7]. Additionally, a post hoc analysis of two placebo-controlled phase 3 trials showed an association between baseline leukocyte count and response to lusotrombopag [14], an association borderline significant also in our univariate analysis. Only determination of circulating TPO levels could clarify whether the pathophysiological determinant of these associations is portal hypertension, liver dysfunction, or hyporegenerative bone marrow.

Regarding safety, in our cohort, de novo PVT occurred in four patients (5%) treated with lusutrombopag, in two cases within one month and in others between 30 and 180 days after the procedures. Actually, it is fair to point out that the absence of following up 3 of 21 procedures may lead to an underestimation of the long-term incidence of PVT.

Notably, previous studies of TPO stimulators have been hampered by pharmacokinetic and safety issues. Eltrombopag, a second-generation stimulator, was withdrawn from studies in the hepatology setting due to a significantly higher incidence of PVT compared with placebo [15]. These concerns are pertinent as TPO-stimulating agents may increase the risk of thrombosis by raising platelet counts and possibly by displacing the overall coagulation process. Importantly, our patients had advanced portal hypertension and active tumor involvement, factors inherently associated with an increased risk of PVT and/or venous thromboembolism [16]. Moreover, the peak platelet count in these patients did not exceed 62,000/μL. Data on the true annual incidence of PVT in cirrhotic patients with portal hypertension and HCC are highly heterogeneous, depending on stage of disease (compensated vs. decompensated), comorbidities and therapeutic interventions, with an annual general estimate ranging from 5% to 15% [17]. A recent systematic review and meta-analysis [17] estimated a pooled overall incidence of PVT in cirrhosis of approximately 10.42 (95%CI = 8.16% to 12.92%) and 4.59 (95%CI = 3.49 to 5.83) per 100 patient years. Of note, this pooled incidence was significantly higher in patients with Child-Pugh B\C (18.34, 95%CI = 10.79% to 27.35%), high-risk esophageal varices (17.88, 95%CI = 13.69% to 22.49%) and ascites (19.91, 95%CI = 11.77% to 29.55%) [18]. In addition, HCC is known to increase the risk of non-neoplastic PVT in cirrhosis [19]. Furthermore, in our study, three out of four thrombosis events occurred after an invasive procedure such as the thermal ablation of HCC, which is known to carry an additional risk of thrombosis [20,21]. Nevertheless, a vigilant observation on larger patient cohorts treated with the drug should certainly be ensured since the risk–benefit profile in these patients, probably determined also from the specific invasive procedure, should be clearly determined.

Another relevant finding emerging from our data, which could not be ascertained in the pivotal trials and is consistent with an earlier Japanese real-world report [7], is that the drug can be safely used repeatedly in the same patient. At the same time, our data showed that treatment failure is likely to be indicative of drug ineffectiveness, as we observed repeated failures in the retreatment of the same patients.

The main strength of this post-marketing surveillance is the evaluation in a real-world context, with inclusion of patients with high heterogeneity, which allowed for the addition of fundamental knowledge for the drug’s use in routine clinical practice. On the other side, the main limitations of this surveillance are inherent to the small sample size and to the observational and real-world study design, which determine the lack of a control arm and of a simultaneous data collection at detailed setpoints (for example, the broad and generalized follow-up). This limitation is particularly relevant for interpreting the incidence of PVT in the best possible way.

In conclusion, this study represents the first real-world evaluation of lusutrombopag in Europe and confirms its efficacy and tolerability profile. The introduction of such agents marks a significant change in the therapeutic landscape for patients with severe thrombocytopenia. Further real-world studies are expected to extend our knowledge on the safety profile of the drug.

## Figures and Tables

**Table 1:** Baseline socio-demographic and clinical characteristics of the REALITY population included in the analysis.

Characteristics	n. 73
Socio-demographics and comorbidities	
Age (years)	66 (56–72)
Sex (female)	23 (32%)
Body mass index (kg/m^2^)	25 (21–28)
Diabetes mellitus	27 (37%)
Liver disease etiology	
HCV	29 (40%)
NASH	16 (21%)
Alcohol	10 (13%)
HBV	11 (15%)
Others	7 (11%)
Child-Pugh Class	
A	45 (63%)
B	20 (28%)
C	6 (9%)
B-blocker treatment	45 (62%)
Ultrasound parameters	
Spleen interpolar diameter (cm)	18(16–20)
Portal vein diameter (mm)	14 (13–17)
History of portal vein thrombosis	14 (19%)
Biochemistry	
Baseline platelets (/μL)	37,000 (33,000–44,000)
Baseline WBC (/μL)	3470 (2495–4391)
Previous TPO-RA use	11 (15%)
Platelets (/μL)	
Baseline (pre-treatment)	37,000 (33,000–44,000)
Pre-procedure (post-treatment)	58,000 (49,000–82,000)
0–48 h after procedure	56,000 (48,500–87,500)
2–30 days after procedure	51,499 (40,000–66,750)

Data are expressed as median and interquartile range or counts and percentages. HCV: hepatitis C virus; NASH: nonalcoholic steatohepatitis; HBV: hepatitis B virus; WBC: white blood cell; TPO-RA: thrombopoietine receptor agonist.

**Table 2:** Socio-demographic and clinical characteristics of patients with a previous history of portal vein thrombosis included in the REALITY population.

Characteristics	n. 14
Socio-demographics and comorbidities	
Age (years)	72 (52–77)
Sex (female)	5(36%)
Body mass index (kg/m^2^)	25 (23–28)
Diabetes mellitus	6 (43%)
Liver disease etiology	
HCV	8 (57%)
NASH	3 (21%)
Alcohol	2 (14%)
HBV	1 (8%)
Child-Pugh Class	
A	9 (64%)
B	4 (28%)
C	1 (8%)
B-blocker treatment	7 (50%)
History of portal vein thrombosis	
Acute portal vein thrombosis	2 (14%)
Portal cavernous transformation	12 (86%)
Ultrasound parameters	
Spleen interpolar diameter (cm)	17 (14–22)
Portal vein diameter (mm)	14 (13–19)
Biochemistry	
Baseline platelets (/μL)	34,000 (26,000–46,500)
Baseline WBC (/μL)	2720 (2300–2920)
Baseline INR	1.4 (1.3–1.6)
Previous TPO-RA use	1 (7%)
Platelets (/μL)	
Baseline (pre-treatment)	34,000 (26,000–46,500)
Pre-procedure (post-treatment)	53,000 (44,500–58,000)
0–48 h after procedure	54,499 (43,750–68,750)
2–30 days after procedure	50,000 (40,000–64,250)

**Table 3:** Factors associated with effective response (platelets ≥ 50,000/μL).

	Univariate			Multivariate *		
	OR	95%CI	*p* Value	OR	95%CI	*p* Value
Age	0.97	0.92–1.02	0.24			
Sex (female)	1.4	0.45–4.88	0.57			
BMI	0.99	0.94–1.03	0.51			
Diabetes mellitus	1.01	0.35–3.10	0.99			
Child Pugh	1.06	0.78–1.51	0.71			
Spleen diameter (cm)	0.74	0.56–0.95	0.02	0.85	0.62–1.01	0.26
Portal vein diameter (mm)	0.95	0.80–1.13	0.52			
Beta blockers treatment	0.70	0.22–2.08	0.54			
Baseline platelets (1000/μL)	1.13	1.06–1.23	<0.001	1.13	1.04–1.26	0.01
Baseline white blood cells (1000/μL)	1.51	1.02–2.43	0.06	1.09	0.74–1.74	0.67
Previous TPO-RA use	0.57	0.15–2.42	0.42			

* Multivariate analysis is adjusted for baseline values of platelets, leucocytes and spleen diameter. TPO-RA: thrombopoietin receptor agonist.

## Data Availability

The original contributions presented in the study are included in the article; further inquiries can be directed to the corresponding author.
